# Deregulated Replication Licensing Causes DNA Fragmentation Consistent with Head-to-Tail Fork Collision

**DOI:** 10.1016/j.molcel.2006.09.010

**Published:** 2006-11-03

**Authors:** Iain F. Davidson, Anatoliy Li, J. Julian Blow

**Affiliations:** 1 School of Life Sciences, University of Dundee, Dundee DD1 5EH, United Kingdom

**Keywords:** DNA

## Abstract

Correct regulation of the replication licensing system ensures that no DNA is rereplicated in a single cell cycle. When the licensing protein Cdt1 is overexpressed in G2 phase of the cell cycle, replication origins are relicensed and the DNA is rereplicated. At the same time, checkpoint pathways are activated that block further cell cycle progression. We have studied the consequence of deregulating the licensing system by adding recombinant Cdt1 to *Xenopus* egg extracts. We show that Cdt1 induces checkpoint activation and the appearance of small fragments of double-stranded DNA. DNA fragmentation and strong checkpoint activation are dependent on uncontrolled rereplication and do not occur after a single coordinated round of rereplication. The DNA fragments are composed exclusively of rereplicated DNA. The unusual characteristics of these fragments suggest that they result from head-to-tail collision (rear ending) of replication forks chasing one another along the same DNA template.

## Introduction

If replication origins fire more than once in a single cell cycle, DNA in the region of the origin is reduplicated, which represents an irreversible genetic change. To prevent this, eukaryotic cells use a replication licensing system that is activated late in mitosis and deactivated prior to S phase (Diffley, 2004, Nishitani and Lygerou, 2004, Blow and Dutta, 2005). Origin licensing comprises the ordered chromatin loading of the origin recognition complex (ORC), Cdc6, Cdt1, and finally the Mcm2-7 complex (the probable replicative helicase) onto origins of replication (Gillespie et al., 2001, Tsuyama et al., 2005). Once a licensed origin initiates, the Mcm2-7 complex travels ahead of the fork, unwinding the DNA to allow access by the replication machinery, thereby leaving behind an unlicensed replication origin.

In metazoans, regulation of the licensing system largely occurs by downregulation of Cdt1 activity during S phase, G2, and early mitosis. Geminin binds to and inhibits Cdt1 throughout this period (McGarry and Kirschner, 1998, Tada et al., 2001, Wohlschlegel et al., 2000, Hodgson et al., 2002, Li and Blow, 2004). In addition, Cdt1 is ubiquitinated and degraded during S phase (Nishitani et al., 2001, Zhong et al., 2003, Thomer et al., 2004, Li and Blow, 2005, Arias and Walter, 2005). In metazoans, overexpression of Cdt1 or downregulation of geminin during S phase and G2 leads to rereplication of chromosomal DNA (Mihaylov et al., 2002, Vaziri et al., 2003, Melixetian et al., 2004, Thomer et al., 2004, Zhu et al., 2004, Arias and Walter, 2005, Li and Blow, 2005, Maiorano et al., 2005, Yoshida et al., 2005).

DNA damage checkpoints sense and respond to DNA damage during S phase and G2 via the intra-S phase checkpoint and the G2/M checkpoint, respectively (Gottifredi and Prives, 2005). It has been shown that deregulation of the licensing system leads to checkpoint activation in humans, *Xenopus laevis*, *Drosophila melanogaster*, and *Saccharomyces cerevisiae* (Mihaylov et al., 2002, Vaziri et al., 2003, Melixetian et al., 2004, Zhu et al., 2004, Archambault et al., 2005, Green and Li, 2005, Li and Blow, 2005, Zhu and Dutta, 2006). This checkpoint induction is associated with the appearance of single-stranded DNA and DNA breaks. It remains unclear how deregulation of the licensing system causes checkpoint activation or DNA damage, though several explanations can be envisaged. One possibility is that the cell can detect aberrant activation of the licensing system in S phase and G2 (a “relicensing sensor”) and responds by activating checkpoints. Another possibility is that the cohesin complex which links sister strands together during S phase and G2 may be unable to encompass more than the two daughter strands that are the product of normal replication, and checkpoints become activated as a consequence. A third possibility is that uncontrolled relicensing and reinitiation of replication lead to unusual DNA structures or unusual types of collision between replication forks, thereby generating double-strand breaks.

Here we investigate the DNA damage and checkpoint activation that occur when the licensing system is deregulated in *Xenopus* egg extracts by addition of recombinant Cdt1. We show that the DNA damage and strong checkpoint activation induced by Cdt1 depends on uncontrolled rereplication. Checkpoint activation correlates with the appearance of DNA fragments whose unusual structure is consistent with their being the product of head-to-tail fork collisions.

## Results

### Activation of Checkpoint Pathways by Recombinant Cdt1

**Figure 1:**
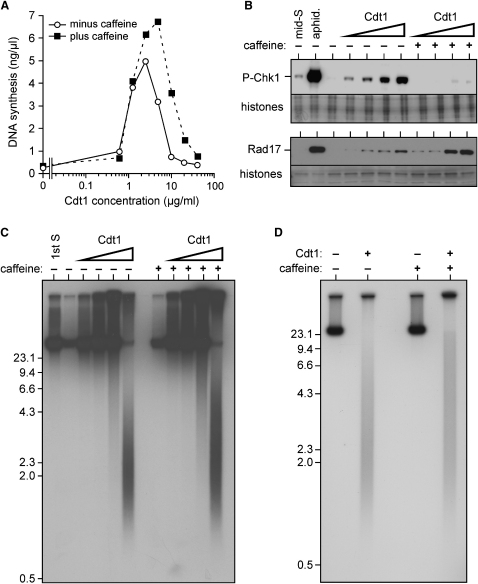
Addition of Cdt1 to Egg Extract Causes Rereplication, DNA Damage, and Checkpoint Activation (A–C) Sperm nuclei were incubated in interphase extract for 90 min; extract was then supplemented with different concentrations of Cdt1 plus or minus 5 mM caffeine and incubated for a further 90 min. (A) Sperm nuclei were added at 5 ng DNA/μl extract. [α^32^P]dATP was added along with Cdt1. After 90 min, rereplication was measured by ^32^P incorporation. (B) Nuclei were immunoblotted for phosphorylated Chk1 (upper panel); chromatin was immunoblotted for Rad17 (lower panel). Final concentrations of Cdt1 were 2.5, 5, 10, or 20 μg/ml. As controls, nuclei were isolated after 40 min sperm incubation by using either untreated extract (mid S) or extract supplemented with 40 μM aphidicolin (aphid.). (C) [α^32^P]dATP was added along with Cdt1. After 90 min, DNA was isolated, separated by neutral agarose gel electrophoresis, and autoradiographed. Final concentrations of Cdt1 were 2.5, 5, 10, or 20 μg/ml. As control, [α^32^P]dATP was added along with the sperm, and DNA was isolated after 90 min (first S). (D) Sperm nuclei were incubated in interphase extract supplemented with [α^32^P]dATP ±20 μg/ml Cdt1 and 5 mM caffeine. After 90 min, DNA was isolated, separated by neutral agarose gel electrophoresis, and autoradiographed. Molecular weight markers (kb) are shown to the side.

To investigate the consequence of deregulating the licensing system, recombinant Cdt1 was added to *Xenopus* egg extracts in G2 of the cell cycle. Consistent with previous reports (Arias and Walter, 2005, Li and Blow, 2005, Maiorano et al., 2005, Yoshida et al., 2005), addition of Cdt1 induced rereplication of DNA (Figure 1A and data not shown). As Cdt1 concentration increased, the amount of rereplication first increased, then fell again. At maximal levels, >50% of the DNA rereplicated, with some DNA undergoing more than one round of rereplication (Li and Blow, 2005 and Figure 1A). The concentration of Cdt1 giving maximum levels of rereplication was typically around 2.5 μg/ml. Addition of caffeine, an inhibitor of the ATM and ATR checkpoint kinases, along with the recombinant Cdt1, increased the amount of DNA replication, consistent with the idea that Cdt1 addition activates checkpoint pathways that in turn suppress further DNA synthesis. Even in the presence of caffeine, however, the amount of DNA synthesis dropped when >5 μg/ml Cdt1 was added, suggesting that these concentrations of Cdt1 cause a structural inhibition of DNA synthesis.

We investigated checkpoint activation by monitoring the phosphorylation of Chk1 and the loading of Rad17 onto chromatin. Chk1 is phosphorylated on Ser345 by ATR in response to a variety of genomic insults (Liu et al., 2000, Guo et al., 2000, Zhao and Piwnica-Worms, 2001). A low level of Chk1 phosphorylation is also seen during undisturbed S phases in *Xenopus* extracts (Figure 1B and data not shown). *Xenopus* Rad17 is loaded onto DNA in response to damage (Stokes et al., 2002), where it is required for the loading of the 9-1-1 complex to chromatin (Jones et al., 2003). Figure 1B shows that Chk1 Ser345 phosphorylation and Rad17 recruitment increased in a Cdt1-dose-dependent manner. As expected, Chk1 phosphorylation, but not Rad17 recruitment, was abolished by caffeine (Stokes et al., 2002, Jones et al., 2003).

To monitor the appearance of damaged DNA, nascent DNA synthesized after Cdt1 addition was labeled with [α^32^P]dATP and was separated by neutral agarose gel electrophoresis. Figure 1C shows that when Cdt1 was added to the G2 extract a smear of nascent DNA fragments appeared. This is consistent with observations that subchromosomal DNA fragments are produced following rereplication in *S. cerevisiae* (Green and Li, 2005) and that double-strand breaks are detectable following rereplication in human cells (Zhu and Dutta 2006). As the concentration of Cdt1 increased, the quantity of these fragments increased while their size decreased; fragments were observed at Cdt1 concentrations as low as 5 μg/ml in some extracts (Figure 1C and see Figure S1 in the Supplemental Data available with this article online). This corresponds well to the concentrations of Cdt1 causing Chk1 phosphorylation. Addition of caffeine to the extract had little effect on the appearance of these DNA fragments. As well as causing checkpoint activation, the appearance of these fragments could also explain the checkpoint-independent inhibition of rereplication that is seen at high Cdt1 concentrations (Figure 1A). To ensure that the effects reported here are not restricted to G2 nuclei, Cdt1 was added to interphase extracts along with sperm, so that rereplication was induced in the first S phase. This also resulted in DNA fragmentation and checkpoint activation (Figure 1D and data not shown).

### DNA Damage and Checkpoint Activation Depend on DNA Rereplication

**Figure 2:**
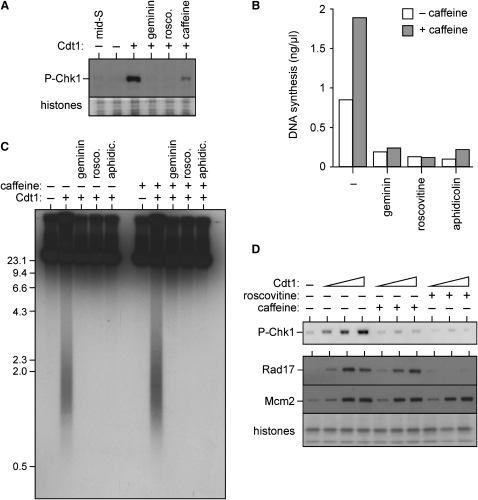
Cdt1-Induced DNA Damage and Checkpoint Activation Requires Rereplication Sperm nuclei were incubated in interphase extract for 90 min to allow a single round of replication; extract was then supplemented with different concentrations of Cdt1 plus or minus caffeine, geminin, roscovitine (2 mM), aphidicolin (100 μM), or [α^32^P]dATP and incubated for 90 min. (A) Nuclei were isolated and immunoblotted for phosphorylated Chk1. As control, sperm nuclei were isolated after 40 min (prior to Cdt1 addition) when they were in mid S phase. Coomassie-stained histones provide a loading control. Cdt1 was added at 20 μg/ml. (B) [α^32^P]dATP was added along with 20 μg/ml Cdt1. After 90 min, ^32^P incorporation into DNA was measured (rereplicative DNA synthesis). (C) [α^32^P]dATP was added along with sperm nuclei, and after 90 min, 20 μg/ml Cdt1 plus or minus inhibitors was added. After a further 90 min, DNA was isolated, separated by neutral agarose gel electrophoresis, and autoradiographed. Molecular weight markers (kb) are shown to the side. (D) Nuclei were isolated and immunoblotted for phosphorylated Chk1 (upper panel), or chromatin was isolated and immunoblotted for Rad17 or Mcm2 (lower panel). Coomassie-stained histones provide a loading control. Final concentrations of Cdt1 were 1.25, 5, or 10 μg/ml.

We next investigated what events were required for recombinant Cdt1 to induce DNA fragmentation and checkpoint activation. The only known biochemical function of Cdt1 is to cooperate with ORC and Cdc6 in the loading of Mcm2-7 onto DNA. This licensing activity is inhibited by geminin. When Mcm2-7 complexes are reloaded onto replicated DNA, they permit the reinitiation of replication, dependent on the activity of cyclin-dependent kinases (CDKs). Replication initiation can be blocked by the CDK inhibitor roscovitine (Luciani et al., 2004). The Chk1 phosphorylation (Figure 2A), rereplication (Figure 2B), DNA fragmentation (Figure 2C), and Rad17 recruitment (Figure 2D) caused by Cdt1 addition were all blocked by the addition of geminin or roscovitine along with Cdt1. Addition of aphidicolin, an inhibitor of replicative DNA polymerases, also abolished the DNA fragmentation (Figure 2C). Taken together, these experiments suggest that the DNA fragmentation and checkpoint activation caused by Cdt1 addition occurs as a direct result of DNA rereplication.

**Figure 3:**
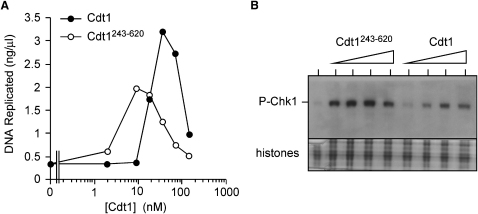
Geminin Titration Is Not Required to Cause Checkpoint Activation Sperm nuclei were incubated in interphase extract (5 ng DNA/μl) for 90 min to allow a single round of replication; extract was then supplemented with different concentrations of full-length Cdt1 or Cdt1^243–620^ and incubated for a further 90 min. Cdt1 constructs were added at 2, 9, 18, 35, 71, 141, and 283 nM, equivalent to 0.16, 0.63, 1.25, 2.5, 5, 10, and 20 μg/ml full-length Cdt1. (A) [α^32^P]dATP was added along with the Cdt1. After 90 min, ^32^P incorporation into DNA, representing rereplicative DNA synthesis, was measured. (B) Nuclei were isolated and immunoblotted for phosphorylated Chk1. Cdt1 constructs were added at 35, 71, 141, and 283 nM. Coomassie-stained histones provide a loading control.

There are two ways that recombinant Cdt1 might be working in these experiments. One possibility is that these effects are directly caused by the recombinant Cdt1 actively relicensing origins; alternatively recombinant Cdt1 might bind and titrate endogenous geminin, thus activating endogenous Cdt1. In order to distinguish these two possibilities, we used a C-terminal fragment of Cdt1 (Cdt1^243–620^) that is able to actively license DNA but is unable to efficiently bind and neutralize geminin (Ferenbach et al., 2005). Cdt1^243–620^ caused both rereplication (Figure 3A) and Chk1 phosphorylation (Figure 3B) at lower concentrations than full-length Cdt1, suggesting that rereplication and checkpoint activation induced by Cdt1 are directly caused by its ability to induce licensing and do not depend on titration of endogenous geminin.

### Uncontrolled Rereplication Is Required For DNA Fragmentation and Strong Checkpoint Activation

**Figure 4:**
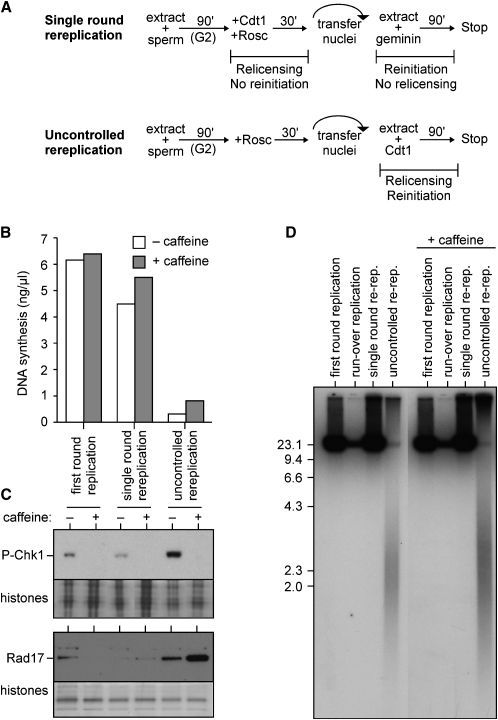
Multiple Rounds of Rereplication Are Required for Cdt1-Induced DNA Damage and Checkpoint Activation (A) Protocol for a single round of rereplication (upper panel) or uncontrolled rereplication (lower panel). (B) [α^32^P]dATP plus or minus caffeine was added to the second extract. As control, roscovitine (0.5 mM) was added to the first extract along with the sperm nuclei (first round replication). (C) Nuclei were immunoblotted for phospho-Chk1 (upper panel), or chromatin was immunoblotted for Rad17 (lower panel). As control, nuclei and chromatin were isolated after incubation for 90 min in the first extract. Coomassie-stained histones provide a loading control. (D) [α^32^P]dATP plus or minus caffeine was added to the second extract. DNA was isolated, separated by neutral gel electrophoresis, and autoradiographed. As control, roscovitine (0.5 mM) was added to the first extract along with the sperm nuclei (first round replication). In a second control, Cdt1 was added to neither extract (run over replication).

We next considered whether checkpoint activation and DNA fragmentation would occur as a consequence of a single round of rereplication or whether multiple rounds of rereplication are required. We devised a protocol, outlined in the upper panel of Figure 4A, to permit Cdt1 to induce only a single round of rereplication. Extracts in G2 were treated with both Cdt1 (to allow relicensing of replicated DNA) and roscovitine (to prevent initiation) for a time sufficient to allow normal levels of Mcm2-7 to be loaded onto DNA (Figure S2); nuclei were then isolated and transferred to fresh extract containing geminin (to block further origin licensing), where the relicensed origins could initiate a single round of rereplication. BrdUTP density substitution experiments confirmed that this resulted in a single round of rereplication (Figure S3A). This protocol was compared with the completely deregulated state, where G2 nuclei were transferred to extract supplemented with recombinant Cdt1 and repeated relicensing and reinitiation occur (lower panel of Figure 4A and Figure S3B).

Using these protocols, we asked whether the inhibition of DNA synthesis by high concentrations of Cdt1 still occurred when only a single round of rereplication was permitted (Figure 4B). Consistent with our previous experiments, only low levels of DNA synthesis occurred when uncontrolled rereplication was driven by 20 μg/ml Cdt1. In contrast, a single round of rereplication induced by 20 μg/ml Cdt1 produced high levels of DNA synthesis. This suggests that the caffeine-insensitive inhibitory effect of high levels of Cdt1 is a consequence of multiple rounds of rereplication.

We then compared the checkpoint activation resulting from a single round of rereplication with that resulting from uncontrolled rereplication using high levels (20 μg/ml) of Cdt1. Uncontrolled rereplication induced much higher levels of Chk1 phosphorylation than occurred after a single round of rereplication or after first round replication (Figure 4C). Rad17 chromatin loading showed similar changes, though the maximal amount of loaded Rad17 varied significantly from extract to extract. Further, fragmented nascent DNA appeared only after uncontrolled rereplication and not after a single controlled round of rereplication (Figure 4D). Therefore, both the DNA fragmentation and strong checkpoint activation caused by Cdt1 occurs as a result of uncontrolled rereplication and not as a result of a single round of rereplication.

### Damage Caused by Cdt1 Is Consistent with Head-to-Tail Fork Collision

**Figure 5:**
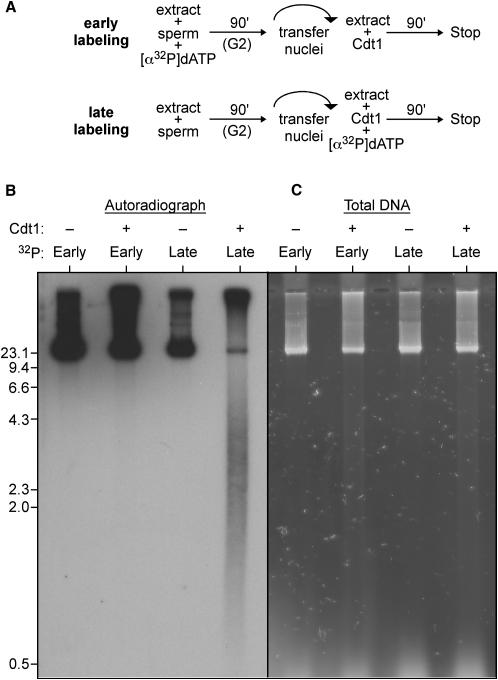
Template DNA Remains Intact during Uncontrolled Rereplication (A) Early labeling protocol (upper panel): only DNA synthesized during the first S phase is labeled with [α^32^P]dATP before uncontrolled rereplication is induced by Cdt1. Late labeling protocol (lower panel): only rereplicated DNA is labeled with [α^32^P]dATP during uncontrolled rereplication induced by Cdt1. (B and C) DNA was subjected to early- and late-labeling protocols as in (A). DNA was separated on neutral agarose gels and either autoradiographed (B) or stained with Sybr Safe to show total DNA (C). Molecular weight markers (kb) are shown to the side.

The experiments above show that, under conditions in which Cdt1 induces checkpoint activation, a smear of nascent DNA fragments is observed on neutral agarose gels. We next investigated how these DNA fragments are generated. We first confirmed that these fragments were specific for Cdt1-induced rereplication and were not generated when DNA replicates normally in egg extract (Figure S4). We next addressed whether all of the DNA became fragmented or just DNA that had undergone multiple rounds of replication. DNA undergoing a first round of replication was labeled with [α^32^P]-dATP, and then unincorporated [α^32^P]-dATP was removed by nuclear transfer (Figure 5A, early labeling). Incubation was then continued in extract plus or minus recombinant Cdt1 and the resultant DNA analyzed by neutral agarose gel electrophoresis and autoradiography. Under these circumstances, Cdt1 caused no fragmentation of the labeled DNA (Figure 5B, ^32^P early). However, when [α^32^P]-dATP was added along with the Cdt1 so that only rereplicated DNA was labeled (Figure 5A, late labeling), a large proportion of the labeled DNA appeared as small fragments (Figure 5B, ^32^P late). This suggests that the fragmented DNA mainly represents DNA undergoing rereplication and does not contain a significant amount of DNA generated by the first (normal) round of replication. Consistent with this idea, almost all the total DNA still migrated at the exclusion limit of the gel, with only a small proportion appearing as fragments (Figure 5C). This specific release of small fragments of rereplicated DNA is exactly what would be expected if uncontrolled rereplication were to cause head-to-tail fork collisions (see Figure 7 below for a model).

**Figure 6:**
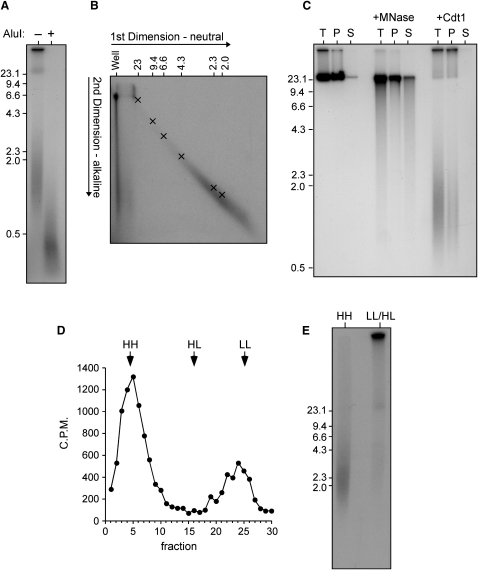
Damage Caused by Cdt1 Is Consistent with Head-to-Tail Fork Collision Sperm nuclei were incubated in interphase extract for 90 min to allow a single round of replication; extract was then supplemented with 20 μg/ml Cdt1 plus [α^32^P]dATP and incubated for a further 90 min. (A) DNA was isolated and incubated plus or minus AluI for 3 hr. Samples were separated by neutral agarose gel electrophoresis and then autoradiographed. (B) DNA was isolated and separated by 2D neutral:alkaline gel electrophoresis. The migration of double-stranded molecular weight markers in the first (neutral) dimension is shown along the top. The migration of double-stranded markers on a parallel 2D electrophoresis is indicated by crosses. (C) Chromatin was pelleted; DNA was isolated from both the pellet fraction (P) and the supernatant fraction (S), and neutral agarose gel electrophoresis was performed. An identical sample was diluted in buffer but not centrifuged to provide total level of DNA (T). As control, sperm nuclei were replicated in the presence of [α^32^P]dATP and then incubated plus or minus micrococcal nuclease (MNase) before being pelleted. (D and E) BrdUTP was added to extract along with Cdt1. After 90 min, DNA was isolated and separated by CsCl equilibrium gradient centrifugation. ^32^P activity across the gradient is shown in (C). HH, HL, and LL show the expected position of heavy/heavy, heavy/light, and light/light DNA, respectively. DNA from fractions 1–10 (HH) and 19–27 (HL/LL) was pooled, separated on a neutral agarose gel, and autoradiographed. Molecular weight markers (kb) are shown to the side.

We next addressed whether the nascent fragments were primarily double- or single-stranded DNA. DNA isolated from an extract supplemented with Cdt1 and [α^32^P]-dATP in G2 was incubated in the presence or absence of the restriction enzyme AluI before being analyzed by neutral gel electrophoresis and autoradiography. Figure 6A shows that the Cdt1-induced DNA fragments were made significantly smaller by AluI, indicating that the fragmented DNA is significantly double stranded. Two-dimensional (neutral:alkali) gel electrophoresis of the nascent DNA fragments was then carried out. Figure 6B shows that the nascent DNA fragments migrated in an arc that comigrated almost exactly with double-stranded marker DNA. This stringent assay indicates that the DNA fragments caused by rereplication are not a heterogeneous mixture of double-stranded, single-stranded, or nicked double-stranded DNA molecules but are almost entirely double stranded. Interestingly, a significant portion of the nascent DNA that migrated as high-molecular-weight DNA under neutral conditions was composed of small fragments, as evidenced by a vertical smear running down the left hand side of the gel in the alkaline dimension. All these features are exactly as would be expected from head-to-tail fork collisions.

We next asked if the DNA fragments remain attached to bulk chromatin in the extract. Chromatin from an extract supplemented with Cdt1 and [α^32^P]-dATP in G2 was pelleted through a sucrose cushion. DNA was isolated from the supernatant and pellet fractions and separated by neutral agarose gel electrophoresis. Figure 6C shows that the fragmented DNA was found exclusively in the pellet fraction, whereas normal chromatin digested with micrococcal nuclease was found in both the pellet and supernatant fractions. This indicates that the fragmented DNA caused by Cdt1 addition remains associated with bulk chromatin in the extract and is only released when the DNA is deproteinized and all chromatin structure is lost.

If the nascent DNA fragments are double stranded but do not contain any DNA present before Cdt1 addition, they must consist entirely of DNA synthesized during the first and second (or higher) rounds of rereplication. To confirm this, BrdUTP and [α^32^P]dATP were added along with 20 μg/ml Cdt1 to an extract in G2; after allowing rereplication to take place, DNA was fractionated on CsCl equilibrium gradients. Figure 6D shows that this revealed an atypical density profile with two broad peaks, one at the density of heavy/heavy DNA (i.e., DNA that had undergone at least two rounds of rereplication) and one smearing up from the density of light/light DNA toward the heavy/light position (i.e., DNA that had undergone only partial rereplication). This profile differs from the more typical density substitution profile found when lower concentrations of Cdt1 are used and DNA fragments are not so evident (Li and Blow, 2005 and Figure S3).

Neutral agarose gel electrophoresis (Figure 6C) revealed that the DNA in the heavy/heavy peak consisted exclusively of small fragments, suggesting that they were created by two or more rounds of rereplication. In contrast, the DNA in the light/light-to-heavy/light peak was predominantly high molecular weight, consistent with the idea that the template DNA remained largely intact. The presence of some DNA fragments in the light/light-to-heavy/light peak may indicate that a small proportion of the template DNA had also been fragmented as a consequence of rereplication. The lack of fully heavy/light DNA is explained by the nascent DNA strands that remain associated with the parental DNA being relatively short (as seen in Figure 6B). These data show that high quantities of Cdt1 cause DNA that has been rereplicated two or more times to be extruded as small fragments, leaving the template DNA largely unchanged.

## Discussion

Previous work had shown that when rereplication is induced by deregulating the licensing system (for example, by overexpressing Cdt1 or removing geminin), checkpoint pathways are activated (Vaziri et al., 2003, Melixetian et al., 2004, Zhu et al., 2004, Archambault et al., 2005, Green and Li, 2005, Li and Blow, 2005, Zhu and Dutta, 2006). We have investigated this by the addition of recombinant Cdt1 to *Xenopus* egg extracts in G2 phase of the cell cycle. We have shown that Cdt1 addition induces Chk1 phosphorylation, the recruitment of Rad17 to chromatin, and the appearance of small nascent DNA fragments. We have shown that none of these effects occur if reinitiation of replication is blocked by coaddition of geminin or roscovitine. We have used Cdt1 mutants to show that both Cdt1-induced rereplication and Chk1 activation can occur independently of the titration of endogenous geminin. These experiments suggest that the major checkpoint response to deregulation of the licensing system occurs as a consequence of the rereplication that this causes. We could find no evidence of a checkpoint system responding directly to Cdt1 activation in the absence of rereplication as has been reported in quiescent mammalian cells (Tatsumi et al., 2006).

We have also shown that strong checkpoint activation and the appearance of small DNA fragments induced by Cdt1 require multiple rounds of rereplication and do not occur after a single round of rereplication. These results suggest that the rereplication of DNA is not itself detected by the checkpoint machinery and argues against models that invoke the inability of the sister chromatid cohesion machinery to cope with more than two sister DNA strands. Consistent with this interpretation, there was no decrease in the production of nascent DNA fragments when Cdt1 was added to extracts immunodepleted of the cohesin complex (data not shown). We have shown that Cdt1 does not induce the loading of excessive quantities of Mcm2-7 onto DNA, which suggests that checkpoint activation is not a consequence of replication forks colliding with a very large number of uninitiated Mcm2-7 complexes. Instead, we show that checkpoint pathways become strongly activated only under conditions in which reinitiation occurs on a replicating template. This could potentially cause a number of unusual DNA structures or unusual types of collision between replication forks, which could in turn lead to double-strand DNA breaks and DNA fragmentation.

**Figure 7:**
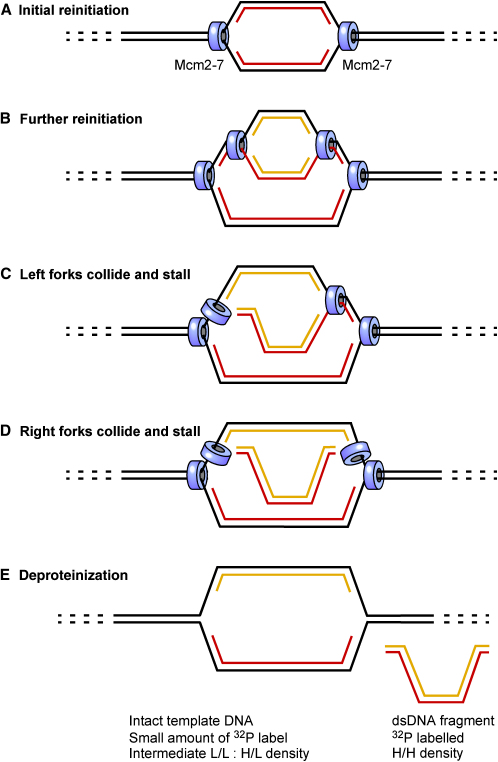
Model for the Creation of DNA Fragments by Head-to-Tail Fork Collision A small segment of chromosomal DNA containing a replication origin is shown. Mcm2-7 is indicated by blue cylinders. (A) Reinitiation at the replication origin forms a replication bubble. (B) A further initiation event forms a second replication bubble whose forks are chasing the forks in the first bubble. (C) The left-moving forks undergo head-to-tail collision, shutting them both down. (D) The right-moving forks undergo head-to-tail collision, shutting them both down. (E) When replication fork proteins are removed from the DNA, a small DNA fragment is released from the chromosomal DNA.

One model that is consistent with all our observations is outlined in Figure 7. When uncontrolled reinitiation occurs, replication forks can chase one another along the DNA (Figures 7A and 7B). Under these conditions, there is the danger of the rear fork running into (rear ending) the front fork, a head-to-tail fork collision (Figure 7C). When this happens, the forks are likely to stall irreversibly, since the Mcm2-7 helicase associated with the rear fork would run out of double-stranded DNA template. Although the helicase associated with the front fork would potentially remain active, it would be separated from the nascent DNA strand by the stalled helicase derived from the rear fork. Such irreversible stalling would explain why rereplication is inhibited by very high concentrations of Cdt1, irrespective of caffeine addition. If both forks in a replication bubble undergo this sort of collision (Figure 7D), the result would be the generation of small double-stranded DNA fragments that would be released on deproteinization of the chromatin (Figure 7E). Consistent with this model, only DNA synthesized after the addition of Cdt1 was found in the fragments, with the template DNA remaining largely intact. The small nascent fragments were fully double stranded, with both strands representing rereplicated DNA. The model also predicts that some nascent DNA would remain associated with the template DNA (Figure 7E), which we observed as intermediate density high-molecular-weight DNA in Figures 6B, 6D, and 6E. Although other explanations are possible, the close agreement between the predictions of the model and our observations argue strongly that some sort of fork collision process was occurring in our experiments. This model is also consistent with results obtained in rereplicating *S. cerevisiae* cells in which reinitiation occurs from only a subset of origins that may reinitiate more than once, and where the reinitiated forks fail to progress far from the origins (Green et al., 2006, Tanny et al., 2006).

In our model, the sooner that reinitiation occurs on a rereplicating template, the closer the two forks will be and so the sooner a collision is likely to occur. This can explain why increasing Cdt1 concentrations decrease the size of the extruded DNA fragments. At the highest Cdt1 concentration we used (20 μg/ml), most forks underwent head-to-tail collision within ∼5 kb. Although fork collision could arise simply as a consequence of natural variation in replication fork rate, this high rate of collision implies that the rear forks typically run faster than the front forks. It is known that nascent DNA directly behind a replication fork is in an “immature form,” being acetylated, less stable, and more susceptible to nuclease degradation than bulk chromatin (Annunziato and Seale, 1983, Cusick et al., 1983, Gasser et al., 1996, Milutinovic et al., 2002). The presence of acetylated histones in nascent DNA persists for up to 20 min following replication (Taddei et al., 1999). It seems plausible, therefore, that the rear forks move faster through this immature chromatin, increasing the probability that they catch up with the front forks. The high rate of fork collision also suggests that reinitiation may occur preferentially at certain origins, similar to what has been described in yeast (Green et al., 2006, Tanny et al., 2006).

In this paper, we have provided evidence for the idea that a major consequence of the deregulation of the replication licensing system is head-to-tail fork collision, leading to irreversible fork stalling and DNA fragment extrusion. We cannot, however, exclude the possibility that DNA damage and checkpoint activation occur by other routes as well. If they do, however, they can account for only a fraction of the total checkpoint activity observed. It is unclear whether cells could recover from having produced these DNA fragments. One possibility is that the DNA fragments could be recombined into the chromosomal DNA to form duplications of the origin-containing DNA. It will be interesting to determine whether this is an important cause of chromosomal change seen in pathological conditions such as cancer that show increased genomic instability.

## Experimental Procedures

### Extract Preparation and Use

*Xenopus* egg extracts were prepared as described (Chong et al., 1997). Cycloheximide (250 μg/ml), 25 mM phosphocreatine, 15 μg/ml creatine phosphokinase, and 0.3 mM CaCl_2_ were added to extract before use. Sperm was added at a final concentration of 5–10 ng DNA/μl extract. Extracts were incubated at 23°C. To measure total DNA synthesis, extracts were supplemented with 50 μCi ml^−1^ [α^32^P]dATP; after incubation, incorporated ^32^P was determined by TCA precipitation and scintillation counting as described (Chong et al., 1997). Aphidicolin (Calbiochem) was used at 40 or 100 μM, roscovitine (Calbiochem) at 0.5 mM or 2 mM, caffeine at 5 mM, and geminin at 100 μg/ml.

### Recombinant Proteins and Antibodies

Full-length his-tagged Cdt1 and his-tagged geminin^DEL^ were produced as described (Ferenbach et al., 2005). Cdt1^243–620^ was a gift from A. Ferenbach. Rabbit anti-XRad17 was a gift from H. Lyndsay (Costanzo et al., 2003). Mouse anti-human Mcm2 was from BD Biosciences, and rabbit anti-human Chk1 P-Ser345 was from Cell Signaling Technology.

### Chromatin Isolation for Immunoblotting

Extract was diluted 25-fold in nuclear isolation buffer (NIB, 50 mM KCl; 50 mM HEPES [pH 7.6]; 5 mM MgCl_2_; 2 mM DTT; 0.5 mM spermidine; 0.15 mM spermine; and 1 μg/ml each leupeptin, pepstatin, and aprotinin) supplemented with 2.5 mM Mg-ATP and 0.1% Triton X-100 and underlayered with 100 μl of this buffer plus 15% sucrose. Samples were centrifuged in a swinging bucket rotor (6000 g, 5 min, 4°C) and the pellet recentrifuged in a fixed-angle rotor (10,000 g, 2 min, 4°C).

### Isolation of Nuclei for Chk1 Immunoblotting

Assays were performed essentially as described (Kumagai et al., 1998). Extract was overlaid on 300 μl Chk1 buffer (50 mM HEPES [pH 7.6], 100 mM KCl, 2.5 mM MgCl_2_, and 1.3 M sucrose). Nuclei were centrifuged in a swinging bucket rotor at 5000 g for 3 min at 4°C. Buffer (200 μl) was removed, and the pellet was resuspended in 500 μl Chk1 buffer. This was then transferred to a fresh tube containing 500 μl Chk1 buffer and the sample recentrifuged as above. Supernatant was removed, leaving 20 μl; 6× SDS PAGE sample buffer was then added.

### Nuclear Transfer

Experiments were performed essentially as described (Blow and Laskey, 1988). Extract was diluted 25-fold in NIB and underlayered with 100 μl NIB containing 15% w/v sucrose. Nuclei were centrifuged in a swinging bucket rotor at 3000 g for 5 min at 4°C. The supernatant was removed and the pellet resuspended in fresh extract such that the fresh extract was diluted no more than 20%.

### Density Substitution

Reactions containing [α^32^P]dATP and 400 μM BrdUTP were stopped in Stop N (20 mM Tris-HCl [pH 8], 200 mM NaCl, 5 mM EDTA, and 0.5% SDS) and incubated with 2 μg/ml RNase for 45 min 37°C, and 200 μg/ml Proteinase K was added for a further 30 min. DNA was extracted (phenol/chloroform, ethanol precipitation) and resuspended in 50 μl TE (10 mM Tris-HCl, 1 mM EDTA [pH 7.5]). Nonincorporated [α^32^P]dATP was removed using a 2.4 ml Sephadex G-50 column. Fractions containing incorporated [α^32^P]dATP were pooled, diluted to 0.5 ml with TE, and mixed with 5.5 ml 109% CsCl in TE. Samples were centrifuged at 36,000 rpm in a 70.1Ti rotor (Beckman) for 40 hr at 20°C. Fractions (200 μl) were collected and radioactivity was measured.

To recover DNA from CsCl fractions after density substitution, density gradient fractions were pooled and dialysed (Upstate Tube-o-Dialyzer Med 15 kDa MWCO) against 1 l TE (pH 8) for 5 hr with frequent buffer changes. DNA was ethanol precipitated in the presence of 1 mg/ml glycogen (Sigma) and resuspended in 20 μl TE.

### Agarose Gel Electrophoresis

DNA was isolated and ethanol precipitated as described above and resuspended in 10 μl TE (for neutral gels) or 10 mM EDTA (for alkaline gels). For alkaline gels, 10 μl 2× alkaline loading buffer (100 mM NaOH, 2 mM EDTA, 2.5% Ficoll, 0.025% bromocresol green) was then added. Alkaline agarose gels were poured in 50 mM NaCl, 1 mM EDTA) and when set were equilibrated >1 hr in alkaline running buffer (50 mM NaOH, 1 mM EDTA). End-labeled λ-phage HindIII markers (New England Biolabs) were used as markers. For the AluI digest, gels were fixed with 7% trichloroacetic acid before autoradiography.

For neutral:alkaline 2D gel electrophoresis, first dimension neutral gel electrophoresis was performed as above. Sample and marker lanes were excised and cast into a second agarose gel equilibrated in 50 mM NaCl, 1 mM EDTA, which was then equilibrated in alkaline running buffer. Electrophoresis of both sample and markers was performed in parallel along with a second marker in the second dimension.

### MNase Chromatin Treatment

Extract was diluted in 200 μl buffer (50 mM NaCl, 50 mM HEPES [pH 7.6], 5 mM MgCl_2_, 10 mM CaCl_2_, 0.1% Triton, 2 mM DTT, 0.5 mM spermidine, 0.15 mM spermine, 1 μg/ml each leupeptin, pepstatin, and aprotinin). Micrococcal nuclease (Roche) was added at a final concentration of 1.4 U/ml and incubated for 10 min at 23°C. Reactions were stopped using 20 mM EDTA. Chromatin was pelleted and DNA isolated as described above.

## Supplemental Data

Document S1. Four Figures
