# Supplementary material for: Deregulated Replication Licensing Causes DNA Fragmentation Consistent with Head-to-Tail Fork Collision
Source: Mol Cell. 2006 Nov 3;24(3):433–43. doi: 10.1016/j.molcel.2006.09.010 (PMC1819398; doi:10.1016/j.molcel.2006.09.010)
Supplement: Document S1. Four Figures [file mmc1.pdf]

Molecular Cell, Volume *24*

Supplemental Data

Deregulated Replication Licensing Causes DNA Fragmentation Consistent with Head-to-Tail Fork Collision

Iain F. Davidson, Anatoliy Li, and J. Julian Blow

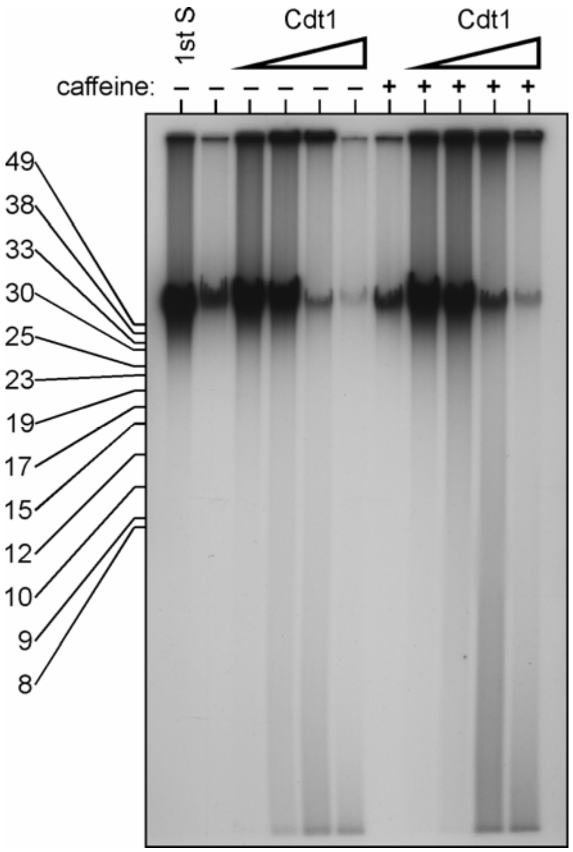

Figure S1. Cdt1 Can Induce DNA Fragmentation at Concentrations as Low as 5 µg/ml
Sperm nuclei were incubated in interphase extract for 90 min to allow a single round of replication; extract was then supplemented with [α^{32}P]dATP and 2.5, 5, 10 or 20 µg/ml Cdt1 plus or minus caffeine. After 90 min, DNA was isolated, separated by neutral agarose gel electrophoresis and autoradiographed. As control, [α^{32}P]dATP was added along with the sperm and DNA was isolated after 90 min (1st S). Electrophoresis conditions used: 0.4% Seachem Gold agarose, 1 V/cm, 20 hours. End-labelled high molecular weight markers (kb) are shown to the side (Invitrogen).

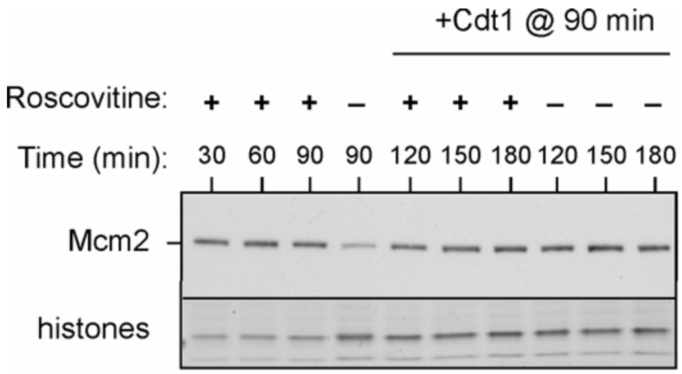

Figure S2. Addition of Cdt1 Does Not Cause Loading of Excessively High Levels of Mcm2
Sperm nuclei were incubated for the indicated times in interphase extract. Chromatin was then isolated and immunoblotted for bound Mcm2. 20 µg/ml Cdt1 was optionally added at 90 min. Roscovitine was optionally added, either at the start of the incubation (for samples without added Cdt1) or at 90 min (for samples with added Cdt1) to prevent any replication-dependent displacement of Mcm2-7 from DNA. Coomassie-stained histones are shown as a loading control.

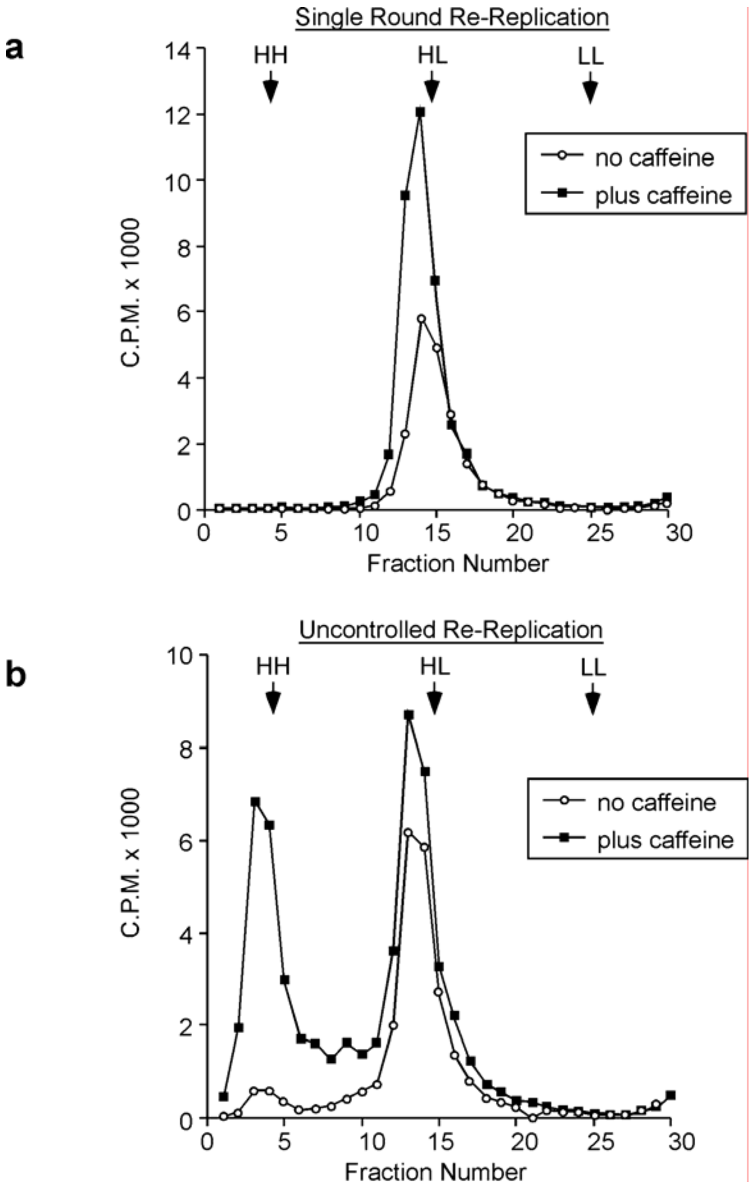

Figure S3. Transfer of Relicensed Nuclei to a Fresh Extract Supplemented with Geminin Results in a Single Round of Rereplication

A, Single round re-replication. Sperm nuclei were incubated in egg extract for 90 min. 5 µg/ml Cdt1 and roscovitine (0.5 mM) were then added, and the incubation continued for a further 30 min. Nuclei were isolated and transferred to a second extract containing geminin, [α^{32}P]dATP and BrdUTP, and incubated for 90 min. DNA was isolated and fractionated on CsCl density equilibrium gradients.

B, Uncontrolled re-replication. Sperm nuclei were incubated in egg extract for 90 min. Roscovitine (0.5 mM) was then added, and the incubation continued for a further 30 min. Nuclei were isolated and transferred to a second extract containing 5 µg/ml Cdt1, [α^{32}P]dATP and BrdUTP, and incubated for 90 min. DNA was isolated and fractionated on CsCl density equilibrium gradients.

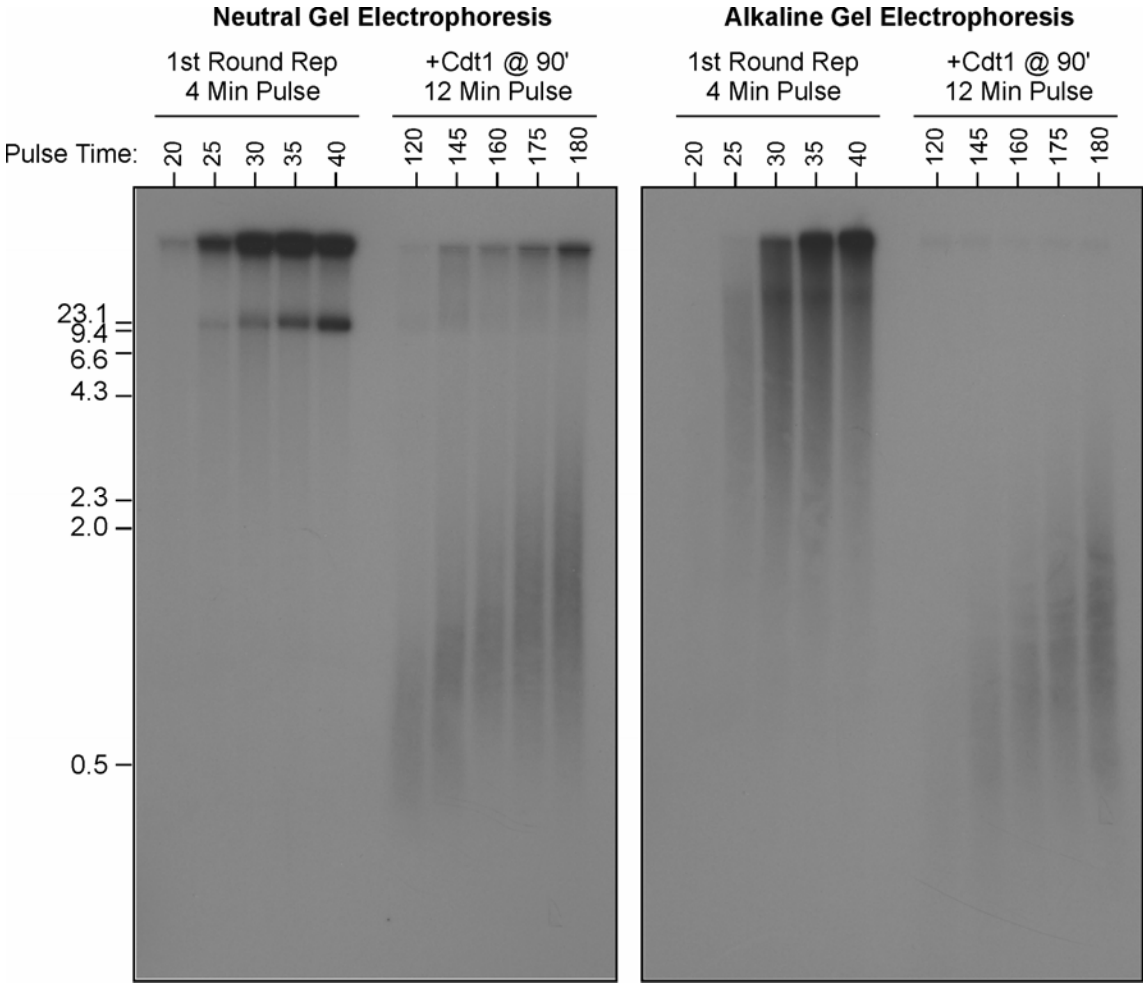

Figure S4. DNA Fragmentation Is Specific for Cdt1-Induced Rereplication and Does Not Occur during Normal DNA Replication

Sperm nuclei were incubated in interphase extract. [α^{32}P]dATP was added at the times indicated and incubated for either 4 minutes (1st round replication) or 12 minutes (re-replication). 20 µg/ml Cdt1 was added at 90 minutes. Samples were split in half; neutral gel electrophoresis was performed on one half, alkaline gel electrophoresis was performed on the other half. Gels were then dried and autoradiographed. The migration of molecular weight markers (in kb) is shown to the left.
